# Elongator Protein 3 (Elp3) stabilizes Snail1 and regulates neural crest migration in *Xenopus*

**DOI:** 10.1038/srep26238

**Published:** 2016-05-18

**Authors:** Xiangcai Yang, Jiejing Li, Wanli Zeng, Chaocui Li, Bingyu Mao

**Affiliations:** 1 State Key Laboratory of Genetic Resources and Evolution, Kunming Institute of Zoology, Chinese Academy of Sciences, Kunming 650223, China; 2 Kunming College of Life Science, University of Chinese Academy of Sciences, Kunming, 650203, China; 3 Center of Molecular Diagnostics, The Affiliated Hospital of KMUST, Medical School, Kunming University of Science and Technology, Kunming 650032, China; 4 China Tobacco Yunnan Industrial Co., Ltd., Kunming, 650024, China

## Abstract

Elongator protein 3 (Elp3) is the enzymatic unit of the elongator protein complex, a histone acetyltransferase complex involved in transcriptional elongation. It has long been shown to play an important role in cell migration; however, the underlying mechanism is unknown. Here, we showed that Elp3 is expressed in pre-migratory and migrating neural crest cells in *Xenopus* embryos, and knockdown of Elp3 inhibited neural crest cell migration. Interestingly, Elp3 binds Snail1 through its zinc-finger domain and inhibits its ubiquitination by β-Trcp without interfering with the Snail1/Trcp interaction. We showed evidence that Elp3-mediated stabilization of Snail1 was likely involved in the activation of N-cadherin in neural crest cells to regulate their migratory ability. Our findings provide a new mechanism for the function of Elp3 in cell migration through stabilizing Snail1, a master regulator of cell motility.

Elongator protein 3 (Elp3) is the catalytic subunit of the Elongator complex that is involved in transcriptional elongation. Elp3 facilitates RNA polymerase II transcription through the acetylation of the N-terminal tail of histone H3. Elp3 contains a C-terminal histone acetyltransferase (HAT) domain that is essential for the ability of the Elongator to acetylate histones i*n vitro*^1^,^2^. In addition to its role in transcriptional elongation, Elp3 is involved in many other biological processes. For example, *Saccharomyces cerevisiae* Elp3 was shown to modulate transcriptional silencing and modulate DNA repair^3^. In *Drosophila* neurons, Elp3-dependent acetylation of Bruchpilot, an ELKS family member, is required for the regulation of the structure of presynaptic densities and neurotransmitter release efficiency^4^. In mouse neurons, Elp3 is found to regulate cell motility and motor-based trafficking via the acetylation of α-tubulin^5^. Interestingly, Elp3 was also reported to be involved in the regulation of cell migration^6^, and depletion of Elp3 leads to the decreased migratory ability of melanoma-derived cells^7^. However, the underlying mechanism of Elp3 to regulate cell migration remains elusive. Elp3 also contains an N-terminal radical S-adenosylmethionine (SAM) binding domain and has been reported to be involved in DNA demethylation^8^.

Neural crest cells have been a classical model to study cell migration *in vivo*^9^. Neural crest cells are induced along the border between the neural and non-neural ectoderm by the interplay of several signaling pathways, including BMP (bone morphogenetic protein), FGF (fibroblast growth factor) and Wnt (Wingless-type protein)^10^. After induction and specification, neural crest cells undergo an epithelial to mesenchymal transition (EMT), delaminate from neighboring tissues and migrate throughout the embryo, producing various derivatives, including craniofacial bone and cartilage, pigment cells, glia and neurons of the periphery neural system, to name a few^11^. The EMT of neural crest cells is regulated by a group of evolutionarily conserved transcription factors, including Snail1 (Snail), Snail2 (Slug), and Twist1^12^,^13^, which modulate cell-cell adhesion and cell polarity to enable the cells to delaminate, accomplish EMT and ensure proper migration.

The zinc-finger transcription repressor Snail1 is a key regulator of EMT that represses the expression of epithelium-specific adherent proteins such as E-cadherin, Muc1, Claudin, and Occludin^14^. However, Snail1 can also act as an activator to stimulate mesenchymal gene transcription^15^. Especially, CREB-binding protein (CBP) has been shown to acetylate Snail1 and prevent repressor complex formation, facilitating the transactivator function of Snail1^16^.

Snail1 is crucial for neural crest migration during embryonic development and has been implicated in the EMT associated with tumor progression^17^,^18^,^19^,^20^. Due to its critical role in development and tumor metastasis, the expression of Snail1 is finely controlled at both the transcriptional and protein levels. The Snail1 protein is labile with a half-life of approximately 25 minutes^21^. The stability of Snail1 is regulated by several ubiquitin E3 ligases, including β-Trcp1, Fbxl14/Fbxl5 and Mdm2^22^. Snail1 phosphorylation in the central domain by glycogen synthase kinase-3β (GSK-3β) promotes β-Trcp-mediated protein ubiquitination and degradation^21^. Fbxl14 and Fbxl5 are two F-box ubiquitin ligases involved in Snail1 degradation. During hypoxia, the expression of Fbxl14 and Fbxl5 is down-regulated, and Snail1 becomes stabilized^22^. Snail1 protein stability is also controlled by inflammatory cytokines through the activation of NF-κB, which then induces the expression of COP9 signalosome 2 (CSN2) to prevent the interaction of Snail1 with GSK-3β/β-Trcp^23^.

Here, we show that knockdown of Elp3 inhibits neural crest cell migration in *Xenopus* embryos. Elp3 binds Snail1 through its zinc-finger domain and inhibits its ubiquitination by β-Trcp. We show evidence that Elp3-mediated stabilization of Snail1 is likely involved in the activation of N-cadherin in neural crest cells to regulate their migratory ability.

## Results

### Elp3 is required for neural crest migration in *Xenopus*

**Figure 1:**
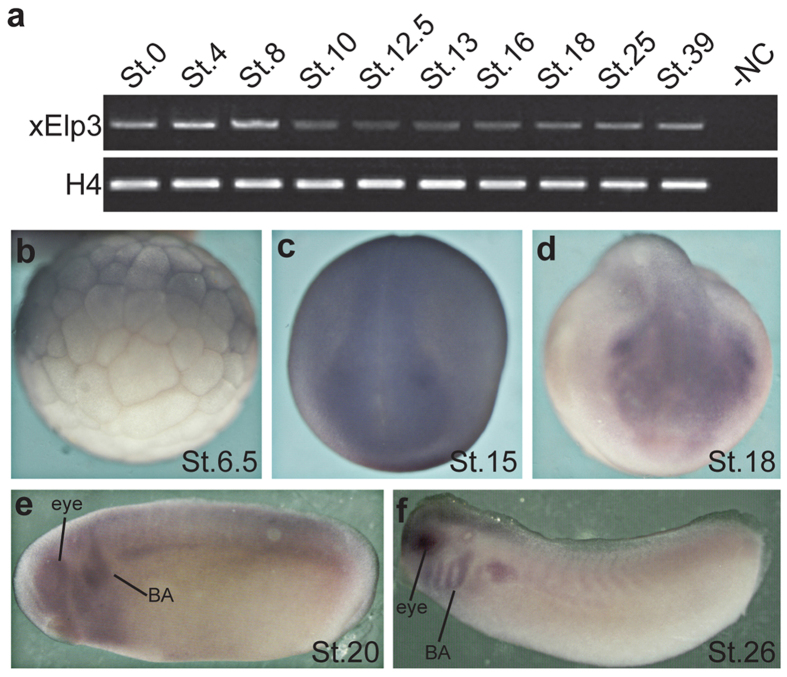
Expression of the Elp3 during early *Xenopus* development. (**a**) RT-PCR analysis of Elp3 expression at different stages (St.0 to St.39). (**b**–**f**). Whole-mount *in situ* hybridization of *xElp3*. *Elp3* transcript is detected in the animal pole at St.6.5 (**b**, lateral view, animal pole to the top). At St.15, *Elp3* is expressed at the anterior neural plate and its border (**c**, dorsal view, anterior to the bottom), and at the late neurulation, *Elp3* is most abundant in cranial neural crest (**d**, frontal view, dorsal to the top). At tailbud stage, *Elp3* is mainly expressed in the branchial arches (BA) and eyes (**e**,**f**).

We investigated the expression pattern of Elp3 in *Xenopus laevis* embryos at various stages. RT-PCR results indicated that *xElp3* is maternally expressed, and the expression is maintained throughout the stages that we examined (Fig. 1a). By *in situ* hybridization, *xElp3* transcripts were detected at the animal pole of stage 6.5 embryos (Fig. 1b). Subsequently, *xElp3* was expressed in the neural plate region (Fig. 1c) and then in the migrating cranial neural crest territory (Fig. 1d). At stages 20 and 26, *xElp3* was detected in the branchial arches, eyes, and prospective brain region (Fig. 1e,f).

**Figure 2:**
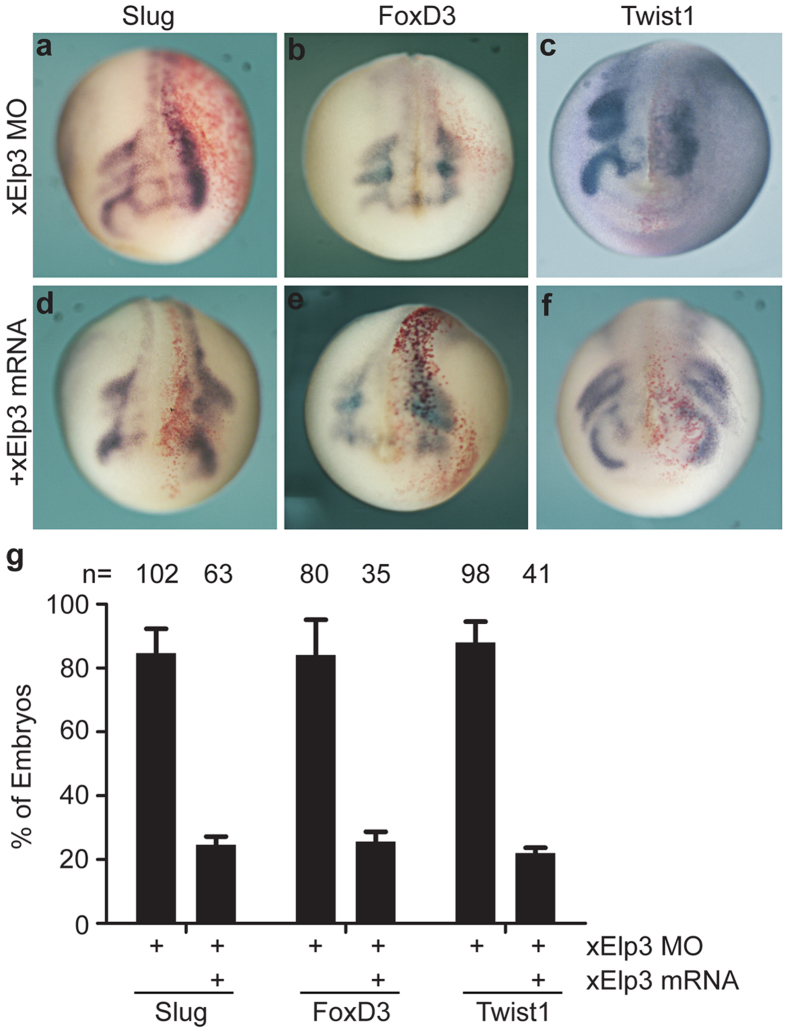
Knockdown of Elp3 inhibits cranial neural crest migration in *Xenopus*. (**a**–**f**) *Elp3* MO (25 ng) with or without *Elp3* mRNA (0.2 ng) was injected into one cell of four-cell-stage embryos, and whole-mount *in situ* hybridization with probes for neural crest markers was processed at St.19–21. *LacZ* mRNA was co-injected to trace the injected sides (stained red on the right sides). (**g**) Percentages of embryos with reduced migration of neural crest as shown in (**a**–**f**). The results are from three independent experiments (error bars represent SDs).

To study the potential role of Elp3 in *Xenopus* neural crest development, we used specific morpholino (MO) to block the expression of endogenous Elp3. The MO efficiently blocked the expression of a GFP reporter mRNA harboring the Elp3 target sequence (data not shown). When probed with the neural crest-specific markers *Slug*, *FoxD3* and *Twist1*, we found that the neural crest cells failed to migrate away from the medial region on the MO-injected side (Fig. 2a–c,g). Co-injection of *Xenopus Elp3* mRNA restored the migratory property of neural crest cells in the Elp3 morphants, confirming the specificity of the Elp3 morpholino (Fig. 2d–f,g).

**Figure 3:**
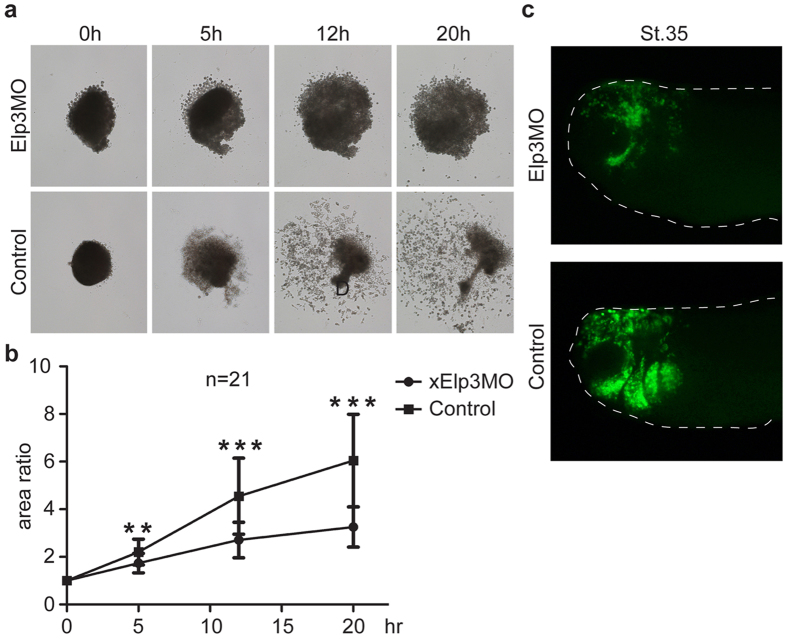
Elp3 is required for cranial neural crest (CNC) migration in explants and transplantation experiments. (**a**) CNC explants were dissected from early neurula embryos, plated on FN (10 μg/ml), and imaged at 0, 5, 12, and 20 h. The surface areas of each explant at every image point were measured, and the ratios of the areas to the values at 0 hour are shown by the line plot (**b**). The data were represented as the means ± standard deviation (SD). **P < 0.01; ***P < 0.001, two-tailed T-test. (**c**) GFP mRNA (100 pg) was injected into one cell at the 4-cell stage with or without Elp3 morpholino (25 ng). At St.15–16, GFP-labeled CNC explants were dissected and grafted into normal host embryos. The status of the fluorescent neural crest was imaged at the tailbud stage (St.35).

We tested the migratory ability of neural crest cells from the Elp3 morphants in the cranial neural crest (CNC) explant assay. When cultured *in vitro*, *Xenopus* CNC cells dissociated and migrated away from the explants on a fibronectin substrate. Indeed, the cells from wild-type neural crest explants migrated from the explants to a considerable distance within a short period (Fig. 3a,b). By contrast, the cells of explants from the Elp3 morphants remained within the explants during the examined period, although they dissociated somewhat (Fig. 3a,b). When GFP-labeled neural crest cells were transplanted into the dorsal region of a host embryo, the grafted cells migrated out efficiently, following prescribed trajectories (Fig. 3c). However, grafted cells from similar regions of the Elp3 morphants mostly stayed where they were transplanted (Fig. 3c), further supporting a role of Elp3 in neural crest migration.

At tadpole stages, the head cartilages in the Elp3 morphants frequently became smaller and malformed while the pigmentation of the embryo was generally normal (data not shown). The modest phenotypic effects on neural crest cell derived tissues suggest the possibility that the effect of Elp3 knockdown might be transient, which delays rather than blocks neural crest migration.

### Elp3 stabilizes Snail1

Snail1 is a key inducer of EMT and regulator of neural crest migration. The N-terminal SNAG domain of Snail1 plays an important regulatory role and mediates protein-protein interaction by mimicking the structure of the histone H3 tail^24^. Because histone H3 is also an Elp3 substrate, we tested whether Elp3 could interact with Snail1 to function in neural crest development.

**Figure 4:**
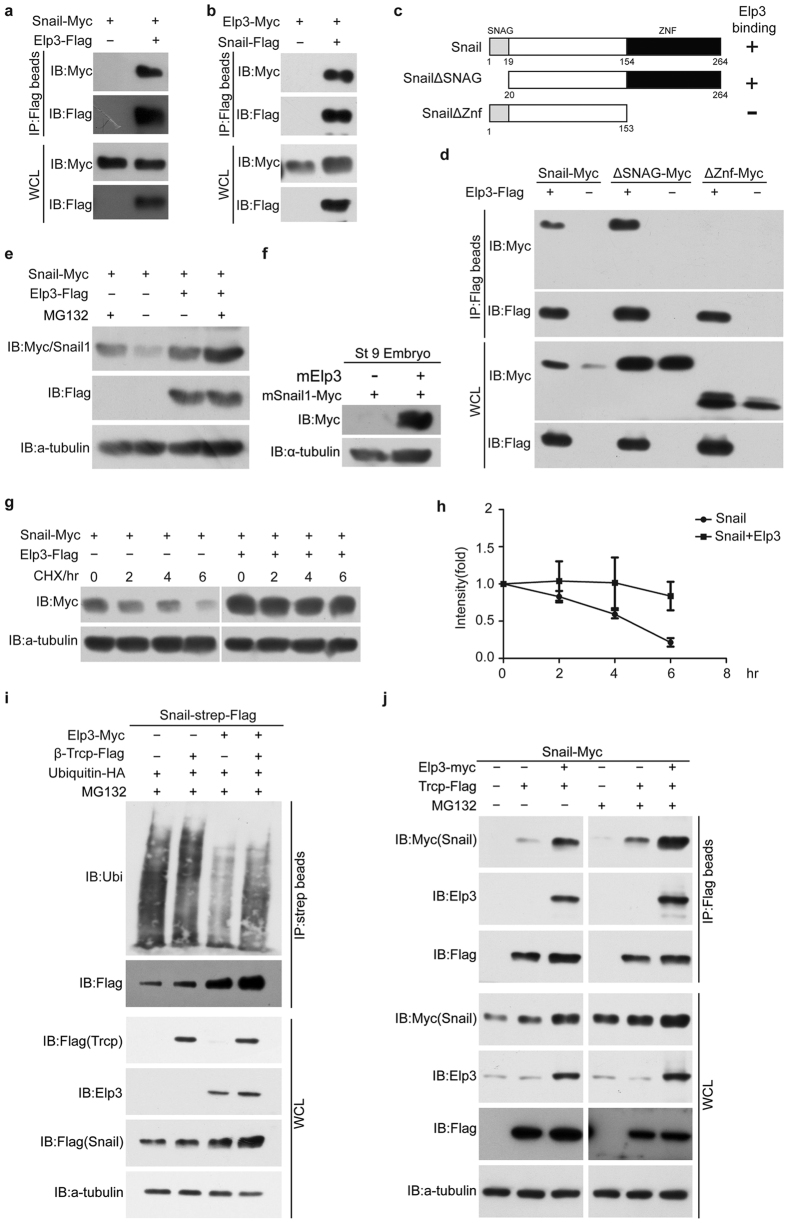
Elp3 interacts with and stabilizes Snail1 through inhibiting its ubiquitination. (**a**,**b**) Co-IP assays showing the interaction between Snail1 and Elp3. (**c**) Schematic representation of the Snail1 deletion constructs. (**d**) In co-IP experiments, Elp3 pulled down full length and ∆SNAG Snail1 efficiently, but not ∆Znf Snail1. (**e**) Co-expression of Elp3 stabilizes Snail1 in 293 cells with or without MG132 treatment (10 μM). The cells were lysed 48 hours after transfection and processed for Western blot analysis. MG132 was added 6–8 hours prior to harvesting. (**f**) Co-expression of mElp3 stabilizes mSnail1 in *Xenopus* embryo. The injected embryos were lysed at stage 9 and processed for Western blot analysis. (**g**,**h**) Elp3 affects the stability of Snail1. HEK293 cells were transiently transfected with the indicated plasmids. At 48 hours post transfection, cycloheximide (CHX) was added to all of the samples, and the cells were then harvested at the timepoints indicated (0 h, 2 h, 4 h, and 6 h). The levels of Snail1 were determined by Western blotting using the anti-Myc antibody (**g**). In all cases, α-tubulin was used as a loading control. The relative levels of Snail1 were quantified densitometrically and normalized against α-tubulin (**h**). The data shown in (**h**) were the average of three independent experiments and represented as the means ± standard error of mean (SEM). (**i**) Elp3 inhibits Snail1 ubiquitination. HEK293 cells were transiently transfected using the indicated plasmids and were treated for 4–5 hours with MG132 before harvesting. Snail1 proteins were immunoprecipitated using Strep-Tactin beads and were probed with an anti-ubiquitin antibody. (**j**) Co-IP experiments showing that Elp3 does not interfere with the Snail1/β-Trcp interaction. HEK293 cells were transiently transfected with the indicated plasmids and then were treated for 6–8 hours with or without MG132 before harvesting. The proteins were immunoprecipitated with anti-Flag M2 beads and then were detected with the antibody against different tags. *WCL, whole cell lysate. IB, immunoblot. IP, immunoprecipitation. CHX, cycloheximide.*

First, we tested whether Elp3 interacts with Snail1. In co-IP assays carried out in mammalian cells, Elp3 could pull down Snail1 and vice versa (Fig. 4a,b). Unexpectedly, the zinc finger domain, not the SNAG domain of Snail1, is indispensable for the interaction (Fig. 4c,d). Snail1 is an unstable protein because it is rapidly polyubiquitinated and degraded through the ubiquitin-proteasomal pathway. Interestingly, co-expression of Elp3 in HEK293 cells or *Xenopus* embryo dramatically increased the protein level of Snail1 (Fig. 4e,f). We also used a series of cycloheximide-based protein chases to test the protein stability of Snail1 with or without Elp3. Snail1 was clearly stabilized in the presence of Elp3 (Fig. 4g,h). These findings indicated that Elp3 could interact with Snail1 and stabilize it.

Next, we tested whether Elp3 attenuates the ubiquitination of Snail1. In HEK293 cells, exogenously expressed Snail1 was heavily poly-ubiquitinated, while co-expression of Elp3 clearly decreased the level of ubiquitinated Snail1 (Fig. 4i), even in the presence of overexpressed β-Trcp (Fig. 4i), a key E3 ubiquitin ligase that mediates the ubiquitination and degradation of Snail1. We then tested whether Elp3 inhibited the β-Trcp-Snail1 interaction to inhibit Snail1 ubiquitination. Intriguingly, we found that β-Trcp binds Snail1 properly in the presence of Elp3, and the three proteins likely formed a ternary complex (Fig. 4j).

**Figure 5:**
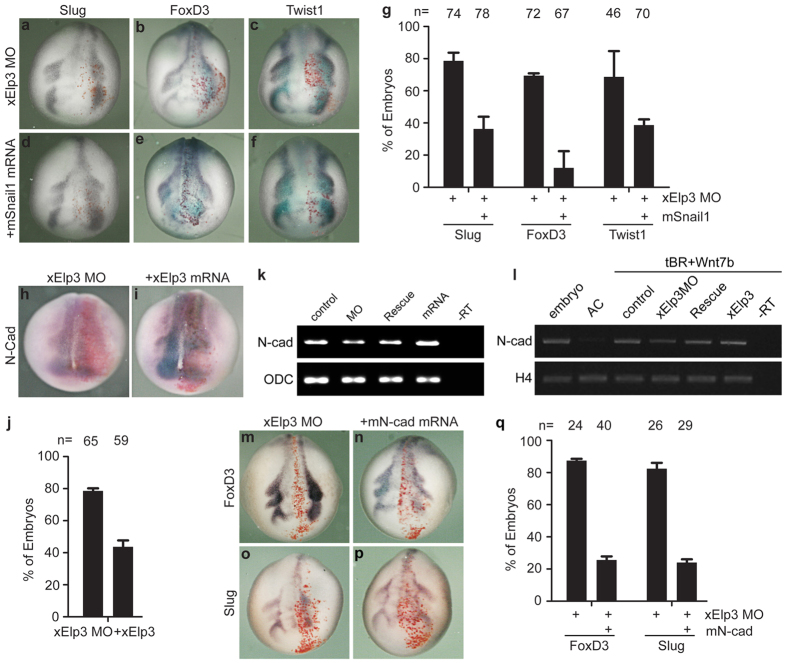
Snail1 or N-cadherin rescues neural crest migration in the Elp3 morphants. (**a**–**f**) Co-injection with *mSnail1* mRNA (0.5 ng) rescues the migration of neural crest cells in the Elp3 morphants. (**g**) Percentages of embryos with reduced migration of neural crest as shown in (**a–f**). The results are from three independent experiments (error bars represent SDs). (**h,i**) Knockdown of *Elp3* by morpholino down-regulates the *N-cadherin* level, while co-injection with *Elp3* mRNA (0.2 ng) restores its expression. (**j**) Percentages of embryos with reduced expression of N-cadherin as shown in (**h**,**i**). The results are from three independent experiments (error bars represent SDs). (**k**) The expression levels of *N-cadherin* in embryos injected with Elp3 morpholino and Elp3 mRNA at St 13. Both cells of the 2-cell stage embryos were injected with Elp3 morpholino (12.5 ng/cell) alone, together with rescue mRNA (0.1 ng/cell), or Elp3 mRNA (0.5 ng/cell) alone. Total RNAs from whole embryos were subjected to RT-PCR analysis for the expression of the indicated genes (cycle numbers: ODC, 24; N-cadherin, 32). (**l**) Elp3 is required for the expression of N-cadherin in induced animal caps. The embryos were injected animally at 2-cell stage with tBR (0.2 ng/embryo) and Wnt7b (0.5 ng/embryo) to induce neural crest fate. Elp3 morpholino and/or mRNA were co-injected as indicated. Animal caps were dissected at stage 9 and harvested when control embryos reached St 19–21. Total RNAs from the animal caps were subjected to RT-PCR analysis for the expression of the indicated genes (cycle numbers: histone H4, 24; N-cadherin, 31). (**m**–**p**) Co-injection with *mN-cadherin* mRNA (0.1 ng) rescues the migration of neural crest cells. *LacZ* mRNA was co-injected to trace the injected sides (stained red on the right sides). (**q**) Percentages of embryos with reduced migration of neural crest as shown in (**m**–**p**). The results are from three independent experiments (error bars represent SDs).

We tested whether overexpression of Snail1 could rescue the migratory ability of neural crest cells in the Elp3 morphants. Indeed, co-injection of mouse *Snail1* (*mSnail1*) mRNA restored the migration of neural crest cells in the Elp3 morphants, as indicated by staining for the neural crest markers *Slug*, *FoxD3* and *Twist1* (Fig. 5a–g). Slug (Snail2) is another member of the Snail family transcription factors involved in neural crest induction and migration^18^,^25^, which shares similar C2H2 zinc finger motifs with Snail1. We tested whether Elp3 also interacts with Slug. Indeed, mElp3 pulled down efficiently xSlug in a co-IP experiment (Supplementary Fig. S1a), and co-expression of xSlug also rescues the phenotype of Elp3 morphants (Supplementary Fig. S1b,c).

Snail1 typically triggers EMT through the transcriptional repression of E-cadherin^26^ and by activating mesenchymal genes, including *N-cadherin*^27^. Interestingly, knockdown of Elp3 reduced *N-cadherin* expression on the injected sides, and this defect could be rescued by supplementing with *xElp3* mRNA (Fig. 5h–j). The result was confirmed by semi-quantitative PCR assay in control and injected whole embryos at stage 13 (Fig. 5k). In animal caps injected with tBR and Wnt7b to induce neural crest fate^28^, knockdown of Elp3 also reduced the expression of *N-cadherin*, which was restored when *xElp3* mRNA was co-injected (Fig. 5l). In addition, overexpression of *mN-cadherin* was also sufficient to rescue the migratory defects in the Elp3 morphants (Fig. 5m–q). By contrast, the expression of *E-cadherin* showed no clear change in the Elp3 morphants (data not shown). Thus, we favor the model that Elp3 binds and stabilizes Snail1, which is required for the transactivation of mesenchymal genes.

## Discussion

The Elongator complex was originally identified in yeast as a transcriptional elongation factor functionally coordinating with RNA polymerase II^29^. The Elongator complex comprises six units, of which Elp3 is the catalytic unit that is indispensable for catalyzing the acetylation reaction. In addition to its role in transcriptional elongation, Elp3 is also involved in cell migration and neurogenesis through the acetylation of α-tubulin^5^,^30^. We showed here that Elp3 is expressed in the neural crest territory and is required for neural crest migration in *Xenopus* embryos. Elp3 interacts with and stabilizes Snail1 through the inhibition of its ubiquitination by β-Trcp. However, in contrast to CSN2, which stabilizes Snail1 through reducing the association between Snail1 and β-Trcp^23^, Elp3 does not work simply through blocking the β-Trcp-Snail1 interaction because the three proteins were found in one complex. It remains possible that Elp3 interferes with the proper spatial positioning or conformation of Trcp and Snail1 required for the ubiquitination reaction.

Snail1 typically triggers EMT through transcriptional repression. E-cadherin is a characteristic marker that is repressed by Snail1 during the initiation of EMT^26^. Snail1 represses E-cadherin transcription by recruiting co-repressors to the promoter and inducing transcriptional silencing^24^,^31^,^32^,^33^. In *Xenopus*, Snail1 is required for both the specification and migration of neural crest cells and has been suggested to work primarily as a repressor in neural crest induction^18^,^19^. However, an increasing body of evidence has indicated that Snail1 can also work as a transcriptional activator during EMT to stimulate mesenchymal genes, including N-cadherin^27^. In cancer cells, Snail1 knockdown decreases while its induced expression increases N-cadherin expression^23^,^24^,^27^,^34^,^35^. However, how exactly Snail1 is involved in N-cadherin regulation remains unknown. In neural crest cells, N-cadherin mediates cell interactions during migration and is required for the contact inhibition of locomotion that is necessary for the collective chemotaxis of neural crest cells^36^. In the Elp3 morphants, the expression of N-cadherin was significantly reduced, a defect that can be rescued by overexpression of *xElp3* (Fig. 5h–k). In addition, the overexpression of *mN-cadherin* is sufficient to rescue the migratory defects of neural crest cells in the Elp3 morphants (Fig. 5m–q). Our data favor a transactivation function of Snail1 to stimulate *N-cadherin* expression in neural crest migration. Interestingly, the acetylation of Snail1 by CBP has been shown to render Snail1 an activator by preventing repressor complex formation^16^. Whether Elp3 works through the acetylation of Snail1 remains to be tested.

## Methods

### Ethics Statement

The care of *Xenopus laevis* (Nasco), *in vitro* fertilization procedure and manipulation of embryos were performed accordingly to standard protocols. All of the animal protocols were approved by the Ethics Committee of Kunming Institute of Zoology, Chinese Academy of Sciences (permit number: SYDW-2006–006).

### Embryo microinjection and whole-mount *in situ* hybridization

*In vitro* fertilization, embryo culture, whole-mount *in situ* hybridization, preparation of mRNA, and microinjection were carried out as described previously^37^. The sequence of the antisense morpholino oligo (MO) for xElp3 is 5′-TTCATGTTGCCCGATGTTCCGCTAG-3′, which targets the 5′ untranslated region plus 5 bases of the coding region and was obtained from Gene Tools. MO and mRNA were injected into the dorsal region of 2- to 4-cell stage embryos. MO was injected at 25 ng/blastomere, and 0.2 ng/blastomere of xElp3 mRNA was coinjected in rescue experiments. For *in situ* hybridization, the probes of *Slug*, *FoxD3* and *Twist1* were used as described previously^37^.

### Plasmid construction

Full-length *Xenopus laevis* and the mouse *Elp3* coding region were obtained by PCR according to sequences in NCBI (accession NM_001095505 and NM_001253812.1) and then were cloned into pCS2^+^ and pCS2^+^-C-Flag/pCS2^+^-N-Myc vectors, respectively. Full-length and truncated mouse *Snail1* constructs were obtained by PCR according to sequences in NCBI (accession NM_011427) and were cloned into a pCS2^+^-N-Myc vector. *Snail1* was also subcloned into the pCS2^+^-C-Flag and pCS2^+^-C-Strep-Flag vectors for co-IP assays and *in vivo* ubiquitination assays, respectively. The mouse *N-cadherin* expression vector was a gift from Dr. Cécile Gauthier-Rouvière (CNRS, France), and the *Trcp-Flag* expression vector is a gift from Dr. Wei Wu (Tsinghua University, China).

### Reverse transcription and polymerase chain reaction (RT-PCR)

To analyze the temporal expression of xElp3 during development, RT-PCR was carried out using whole embryos at different developmental stages. The primers used were as follows: xElp3: 5′- TACCTGAGGATTACAGAGAC-3′ and 5′- CTCTCTCCAGTCCCATGTT-3′. The expression levels of *N-cadherin* in whole embryos or animal caps were also monitored by RT-PCR to reveal the effect of Elp3 using primers as published^38^. H4 or ODC was used as a loading control^38^,^39^.

### *In vitro* cranial neural crest (CNC) migration assay and CNC transplantation

The *in vitro* CNC migration assay and CNC transplantation experiments were carried out as described previously^40^,^41^. CNC explants were dissected from stage 15–16 embryos and were plated onto fibronectin (10 μg/ml in PBS)-coated 96-well plates in Danilchik media (53 mM NaCl, 11.7 mM Na_2_CO_3_, 4.25 mM potassium gluconate, 2 mM MgSO_4_, 1 mM CaCl_2_, 17.5 mM Bicine, 1 mg/ml BSA; pH 8.3), and images were captured every 4–5 hours for up to 20 hours using an Olympus IX83 inverted microscope. The relative surface areas of the explants were measured using ImageJ.

For grafting experiments, the cranial neural crest explants were dissected from GFP-labeled donor embryos and were inserted isotopically and isochronically into unlabeled host embryos from which the cranial neural crest tissue was removed. The grafted embryos were allowed to heal in 1× MBS media (88 mM NaCl, 1 mM KCl, 2.5 mM NaHCO_3_, 0.3 mM CaNO_3_, 0.41 mM CaCl_2_, 0.82 mM MgSO_4_, 15 mM HEPES (pH 7.6) with antibiotics) for 30 min-3 hours at room temperature and then were transferred to 0.1× MBS before being imaged at the late tailbud stage^42^.

### Coimmunoprecipitation (co-IP) assay and immunoblotting

For Snail1/Elp3 and Snail1/Elp3/Trcp co-IP, HEK293 cells were transfected to 6-well plates with the indicated plasmids. At 48 hours post-transfection (treatment for 6–8 hours with or without MG132 before harvest), the cells were lysed in 700 μl of lysis buffer (50 mM Tris-HCl at pH 7.4, 150 mM NaCl, 5 mM EDTA at pH 8.0, and 1% Triton X-100) containing a protease inhibitor mixture (Roche) for 30 min on ice. Following centrifugation at 14000 rpm for 15 min at 4 °C, the supernatant was incubated with Flag-M2 beads (Sigma) at 4 °C for 4 hours and then washed three times with the lysis buffer at 4 °C for 5 min. The bound proteins were eluted with SDS-loading buffer at 95 °C for 10 min and then were subjected to SDS-PAGE and Western blot analysis. The antibodies used were as follows: anti-Flag (M2; Sigma), anti-Myc (Sigma), and anti-Elp3 (Sigma). Horseradish peroxidase-conjugated anti-mouse or -rabbit IgG (Pierce) was used as the secondary antibody.

### *In vivo* ubiquitination assays

*In vivo* ubiquitination assays were carried out as described previously^43^. HEK293 cells were transfected with the HA-ubiquitin construct together with the indicated plasmids. Next, 10 μM of the proteasomal inhibitor MG132 was added 4–5 hours prior to harvesting. Forty-eight hours after transfection, the cells were harvested and lysed in SDS lysis buffer (50 mM Tris-HCl at pH 6.8, 1.5% SDS) at 95 °C for 15 min. Following a 10-fold dilution of the lysate with EBC/BSA buffer (50 mM Tris-HCl at pH 6.8, 180 mM NaCl, 0.5% NP-40 and 0.5% BSA) plus protease inhibitors (Roche), the cell lysates were immunoprecipitated with Strep-Tactin beads (iBA).The bound proteins were eluted using Laemmli sample buffer at 95 °C for 5 min. Western blot analysis was performed using an anti-ubiquitin antibody (Santa Cruz Biotechnology). The membrane was then stripped and reprobed with the other indicated antibodies.

## Additional Information

**How to cite this article**: Yang, X. *et al.* Elongator Protein 3 (Elp3) stabilizes Snail1 and regulates neural crest migration in *Xenopus*. *Sci. Rep.* **6**, 26238; doi: 10.1038/srep26238 (2016).

## Supplementary Material

Supplementary Information
