# Supplementary material for: Elongator Protein 3 (Elp3) stabilizes Snail1 and regulates neural crest migration in Xenopus
Source: Sci Rep. 2016 May 18;6:26238. doi: 10.1038/srep26238 (PMC4870573; doi:10.1038/srep26238)
Supplement: Supplementary Information [file srep26238-s1.pdf]

Supplemental Information

Elongator Protein 3 (Elp3) stabilizes Snail1 and regulates neural crest migration in *Xenopus*

Xiangcai Yang, Jiejing Li, Wanli, Zeng, Chaocui Li, Bingyu Mao

Figure S1

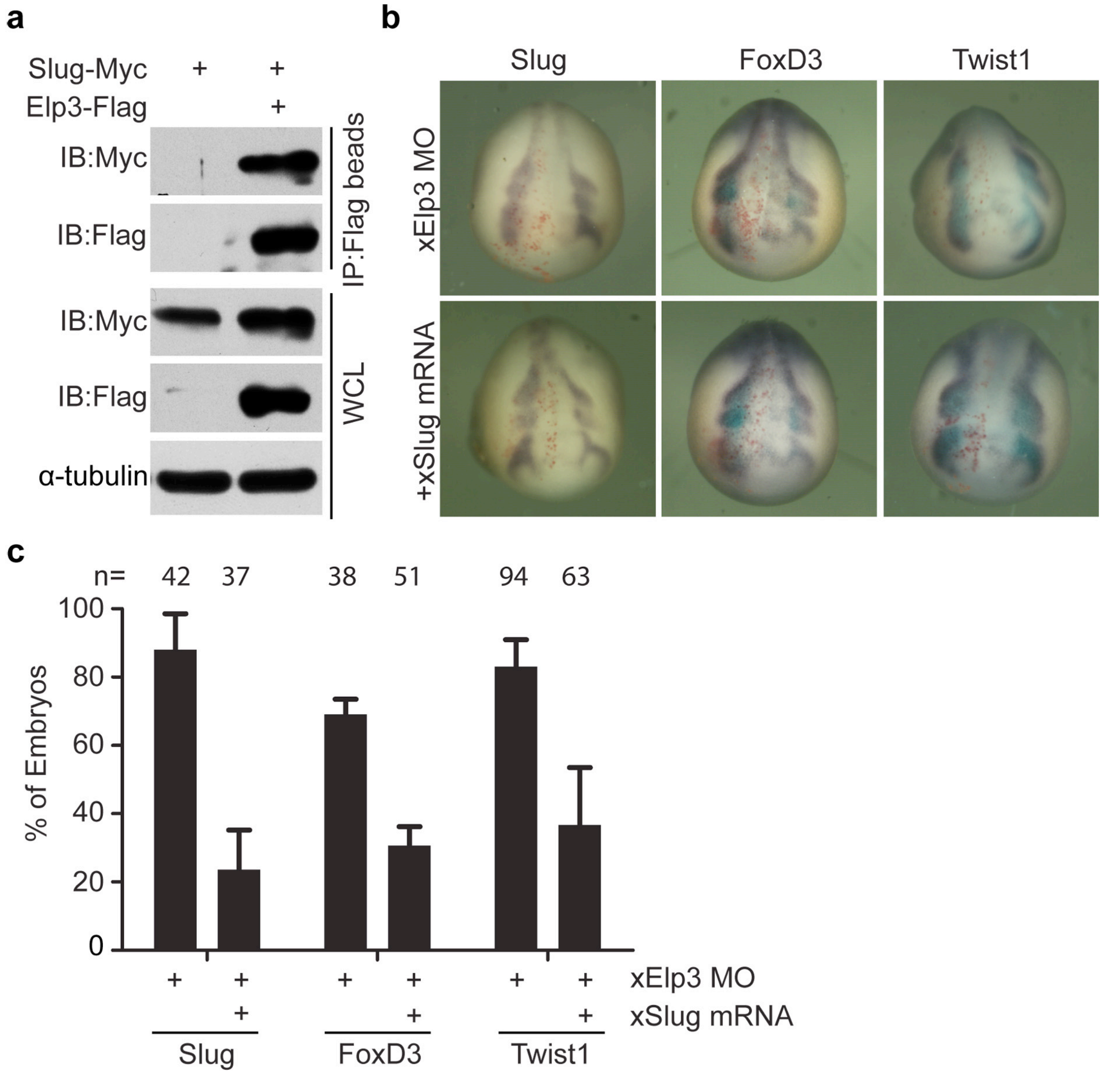

Figure S1 Elp3 interacts with xSlug and xSlug rescues neural crest migration in the Elp3 morphants. (**a**) mElp3 pulls down xSlug in co-IP experiment when co-expressed in HEK 293 cells. *WCL, whole cell lysate. IB, immunoblot. IP, immunoprecipitation.* (**b**) Co-expression of

xSlug mRNA (150 pg/cell at 4-cell stage) in Xenopus embryo restored neural crest migration in Elp3 morphants. Whole-mount in situ hybridization with probes for neural crest markers was processed at St.19-21. LacZ mRNA was co-injected to trace the injected sides (stained red on the left sides). (**c**) Percentages of embryos with reduced migration of neural crest as shown in (**b**). The results are from three independent experiments (error bars represent SDs).
